# Reduction of nosocomial pneumonia after major burns by trace element supplementation: aggregation of two randomised trials

**DOI:** 10.1186/cc5084

**Published:** 2006-11-02

**Authors:** Mette M Berger, Philippe Eggimann, Daren K Heyland, René L Chioléro, Jean-Pierre Revelly, Andrew Day, Wassim Raffoul, Alan Shenkin

**Affiliations:** 1 Department of Adult Intensive Care Medicine & Burn Centre, Centre Hospitalier Universitaire Vaudois (CHUV), Rue du Bugnon 46, 1011 Lausanne, Switzerland; 2 Clinical Evaluation Research Unit, Kingston General Hospital, 76 Stuart Street, K7L 2V7 Kingston, Ontario, Canada; 3 Plastic and Reconstructive Surgery, Department of Surgery, CHUV, Rue du Bugnon 46, 1011 Lausanne, Switzerland; 4 Department of Clinical Chemistry, University of Liverpool, Duncan Building, Daulby Street, L69 3GA Liverpool, UK

## Abstract

**Introduction:**

Nosocomial pneumonia is a major source of morbidity and mortality after severe burns. Burned patients suffer trace element deficiencies and depressed antioxidant and immune defences. This study aimed at determining the effect of trace element supplementation on nosocomial or intensive care unit (ICU)-acquired pneumonia.

**Methods:**

Two consecutive, randomised, double-blinded, supplementation studies including two homogeneous groups of 41 severely burned patients (20 placebo and 21 intervention) admitted to the burn centre of a university hospital were combined. Intervention consisted of intravenous trace element supplements (copper 2.5 to 3.1 mg/day, selenium 315 to 380 μg/day, and zinc 26.2 to 31.4 mg/day) for 8 to 21 days versus placebo. Endpoints were infections during the first 30 days (predefined criteria for pneumonia, bacteraemia, wound, urine, and other), wound healing, and length of ICU stay. Plasma and skin (study 2) concentrations of selenium and zinc were determined on days 3, 10, and 20.

**Results:**

The patients, 42 ± 15 years old, were burned on 46% ± 19% of body surface: the combined characteristics of the patients did not differ between the groups. Plasma trace element concentrations and antioxidative capacity were significantly enhanced with normalisation of plasma selenium, zinc, and glutathione peroxidase concentrations in plasma and skin in the trace element-supplemented group. A significant reduction in number of infections was observed in the supplemented patients, which decreased from 3.5 ± 1.2 to 2.0 ± 1.0 episodes per patient in placebo group (*p *< 0.001). This was related to a reduction of nosocomial pneumonia, which occurred in 16 (80%) patients versus seven (33%) patients, respectively (*p *< 0.001), and of ventilator-associated pneumonia from 13 to six episodes, respectively (*p *= 0.023).

**Conclusion:**

Enhancing trace element status and antioxidant defences by selenium, zinc, and copper supplementation was associated with a decrease of nosocomial pneumonia in critically ill, severely burned patients.

## Introduction

Although the incidence of non-pulmonary infections has decreased in severely burned patients [1], nosocomial pneumonia, including ventilator-associated pneumonia (VAP), remains an important cause of morbidity and mortality [2,3]. During critical illness, oxidative stress is proportional to the severity of the condition [4] and is particularly marked in burned patients [5,6]. Patients with major burns suffer acute early trace element depletion caused by the large exudative trace element losses (selenium, copper, and zinc), which persist until wound closure [7]. Oxidative stress is worsened by these trace element deficiencies, particularly of selenium, which is essential for activity of glutathione peroxidase (GSHPx), a major antioxidant selenoenzyme among selenoproteins [8]. Selenium and zinc deficiencies have also been linked to impaired immune response, and one underlying mechanism is probably the inactivation of GSHPx [9]. During VAP, oxidative stress has been shown to increase early as a consequence of activation of the inflammation cascades [10]. The pathophysiology of VAP includes, among other factors, impaired host-defence mechanisms [1,11]. A series of studies suggest that correcting trace element deficiencies and enhancing the antioxidant capacity in critically ill patients by trace element supplementation reduce morbidity [12,13]. A recent meta-analysis of 11 trials suggested that selenium supplementation is associated with better clinical outcome and a reduction of intensive care unit (ICU) mortality [14].

We conducted two consecutive randomised trials of trace element supplementation in severely burned patients [15,16] after having demonstrated large exudative losses of these trace elements [7,17]. The purpose of the first study was to investigate the impact of trace element supplements on overall morbidity and on immune function, while monitoring the plasma concentration [15]. In 20 patients, pulmonary infections during the first 30 days were apparently reduced, with an improved immune response and an associated reduction of length of stay normalised for percentage of burned body surface area (BSA). The second study was similar in design but focused on wound healing and tissue levels of the trace elements and antioxidant enzymatic activity [16]. We observed a reduction of pulmonary infectious complications, a normalisation of plasma GSHPx activity, increased tissue selenium and zinc concentrations, an improved wound healing, and a similar reduction of normalised length of stay.

However, neither trial was adequately powered to analyse nosocomial infections. The present study combines the data from the two trials and explores the effect of trace element supplementation on nosocomial (or ICU-acquired) pneumonia.

## Materials and methods

### Study design

Two prospective, randomised, double-blind, placebo-controlled, trace element (copper, selenium, and zinc) supplementation studies were conducted consecutively at the burn unit of the department of adult critical care medicine (ICU) in a tertiary university hospital (Centre Hospitalier Universitaire Vaudois [CHUV], Lausanne, Switzerland). Study periods were from 1993 to 1996 and from 1998 to 2003. The two trials were aggregated: although infectious endpoints were identical, their metabolic endpoints differed slightly (Table 1). The ethical committee approved both studies, and written informed consent was obtained from the patients or their next-of-kin.

### Patient population

Inclusion criteria were thermal burns involving more than 20% of BSA in patients who were 16 to 65 years old. Patients with chronic renal failure (creatinine >150 μmol/l), chronic liver disease with liver insufficiency, pregnancy, morbid obesity (body mass index >35), or imminent death (do-not-resuscitate orders within 48 hours of injury) were excluded.

### Supplement administration

The patients were randomly assigned within 12 hours of admission to receive either trace element supplements or placebo by a central intravenous line: the patients were allocated to the treatments using a random list with a block size of 4. The trace elements were provided as sodium selenite, copper gluconate (provided as powder), and zinc gluconate (Selenite^®^, Zinc^®^, Nonan^®^; Laboratoires Aguettant, Lyon, France). Blinding was carried out by the CHUV pharmacist, and the trace element or saline solutions were undetectable on inspection (colour, labelling). Patients, nurses, doctors, and investigators were blinded.

### Outcome measures

The objectives of these two studies regarding infectious complications were identical. Infectious complications were prospectively surveyed during the first 30 days after admission by a trained research nurse and validated by an investigator before unblinding (MMB). The type and the time of onset of infections according to predefined criteria (see below) were evaluated and recorded [18], as were the type, the timing, and the reason for administration of all antimicrobial agents.

Specific variables of burn severity (total burned BSA, inhalation injury, and Ryan score) [19], demographic data, length of mechanical ventilation, and length of ICU stay were recorded. Length of ICU stay is reported as days per percentage of BSA burned. Severity of the initial condition was assessed by SAPS II (Simplified Acute Physiology Score) [20]. Blood samples were collected according to the primary endpoints. In the second trial, skin biopsies were collected. Copper, zinc, and selenium in plasma, and zinc and selenium in skin were determined in duplicate by inductively coupled plasma mass spectrometry (Plasmaquad 3 Inductively Coupled Plasma Mass Sectrometry (ICP-MS); VG Elemental, Winsford, Cheshire, UK). Plasma GSHPx was determined by the Ransel method (Randox Laboratories Ltd., Belfast, UK).

Pneumonia was defined as the combination of systemic inflammatory response syndrome (SIRS) (temperature >38°C, tachycardia, and leucocyte count >12,000 cells per mm^3^) with a new infiltrate on the chest radiograph (or progression of an existing infiltrate), a new or persisting hypoxaemia (partial pressure of oxygen [PaO_2_] <70 mmHg with air or PaO_2_/FiO_2 _[fraction of inspired oxygen] ratio <200), and purulent sputum or tracheal secretion. Microbial confirmation, obtained by culture of bronchoscopic bronchoalveolar lavage (BAL) or blind non-bronchoscopic mini-BAL, was required in all mechanically ventilated patients. Pneumonia occurring during the first 48 hours was considered as community- or trauma-acquired and also to be less susceptible to benefit from the supplementation and was scored as early pneumonia [21]. Nosocomial pneumonia was defined as a pneumonia occurring 48 hours or more after admission. VAP was a nosocomial pneumonia acquired more than 48 hours after endotracheal intubation while on mechanical ventilation.

### Statistical analysis

Data from both trials were aggregated based on similarity of supplementation and on identical surveillance methods of the infectious complications. Patient characteristics were compared between studies by the *t *test for continuous variables and by the Fisher exact test for categorical variables. All other analyses were stratified by study. The Mantel-Haenszel test was used to compare binary variables, the exact stratified Cochran-Armitage trend test was used to compare the number of infections per patient, and the stratified log-rank test was used to compare time-to-event and duration variables. All time-to-event (duration) variables are presented as medians with ranges, and a Kaplan-Meier curve is presented to compare time to first episode of nosocomial pneumonia between groups.

## Results

Forty-one patients, 42 ± 15 years old, who were burned on 46% ± 19% of BSA were included (Table 2). The characteristics of these patients, including the severity of burns and the initial need for supporting organ failures, were similar across studies and between the supplementation (*n *= 21) and placebo (*n *= 20) recipients in each study. Plasma selenium and GSHPx concentrations were normalised and significantly higher in the supplemented patients after day five, and plasma zinc was higher from day ten, as were the skin selenium and zinc concentrations on day 20 (*p *< 0.02).

Among clinical endpoints (Table 3), mortality and requirement for mechanical ventilation did not differ significantly. The number of days of antibiotherapy was significantly lower in the supplemented group (*p *= 0.021). The length of stay was significantly reduced in the supplemented patients, with a median of 0.63 days versus 0.99 days per percentage of burned BSA (*p *= 0.002).

**Figure 1:**
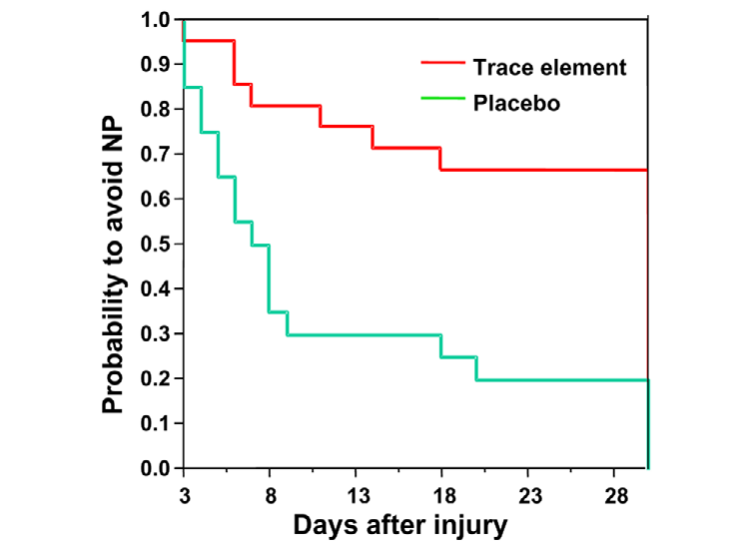
Kaplan-Meier plot of the first episode of nosocomial pneumonia (NP). Red line represents trace element-supplemented group; green line represents placebo group. *P *= 0.002 by stratified log-rank test.

All patients presented at least one episode of infection (Table 4). However, the number of infectious complications was lower in the supplemented group, this decrease being due to a lower number of nosocomial pneumonias (a mean of 0.33 episodes per patient in the supplemented group versus 1.55 episodes per patient in the placebo group, *p *< 0.001; Table 4). The difference remained significant when only the first episode of nosocomial pneumonia was considered (Figure 1). VAP was also significantly less frequent (*p *= 0.001). Finally, in the supplemented group, significantly fewer patients experienced recurrent pneumonia (that is, new distinct pneumonia with different microorganisms and new pulmonary infiltrates occurring after a first episode whether early or nosocomial). Wound, bloodstream, and urinary infections did not differ between the groups.

## Discussion

Our data show a marked and significant reduction in nosocomial pneumonia and VAP in a cohort of severely burned patients in whom trace element supplementation enhanced the antioxidant defences. Indeed, plasma selenium and GSHPx concentrations were significantly higher in the supplemented group after day five, as was zinc after day 10 [15,16].

VAP has been shown to be associated with early oxidative stress as assessed by a decline in GSHPx activity in the plasma and in the alveolar fluid [10]. Indeed, selenium is essential for the activity of the various types of GSHPx, and restoring selenium plasma concentrations has been shown to restore their activity and the antioxidant status [8,22]. It has also been suggested that, in critically ill patients with severe SIRS, selenium supplementation may prevent acute renal failure because oxidative stress is implicated in its pathophysiology [12]. In addition, trace element supplementation is likely to improve neutrophil, macrophage, and lymphocyte function in severely burned patients; in truth, we observed higher neutrophil counts in the supplemented patients of the first trial [15]. Therefore, the reinforcement of antioxidant and metabolic status by trace element supplementation is a likely underlying pathophysiologic mechanism, explaining the prevention of nosocomial pneumonia we observed in the supplemented patients.

There are some limitations to the interpretation of these results. There are minor differences between the designs of the two studies we merged: (a) doses of and length of administration of supplements of selenium, of copper, and of zinc were higher in the second trial, (b) the primary metabolic and nutritional endpoints differed slightly (Table 1), (c) the studies were consecutive in time, which might have been associated with modest changes in general therapeutic procedures, and (d) despite the highly significant results, the number of patients was low. Aggregation of the data may therefore be criticised, even though the characteristics of the patients were similar in both studies and were well-balanced across treatment arms. However, both studies were double-blind, placebo-controlled, and explored the same therapeutic concepts. In addition, the type of infection surveillance, including definitions for infectious complications, was identical. Accordingly, the differences are unlikely to have significantly influenced the rate of infectious complications.

The absence of impact on cutaneous infections raises questions. Why was the lung protected while the skin was not? Various hypotheses may explain this difference. First, because the intravenous infusion of the supplements in a central venous line will first pass through the lung, the concentrations of trace elements could be higher in the lung than in the skin; this hypothesis is supported by the delayed increase of the selenium content of the skin. Indeed, in the biopsies of the burned skin, selenium and zinc concentrations were similar in both groups on days three and ten and increased significantly on day 20 in the supplemented patients [16]. The values on day 20 correspond to a normalisation of skin content compared with healthy volunteers (unpublished data), suggesting that cutaneous deficit might persist despite supplementation for two to three weeks. Second, the pathophysiology of pneumonia may differ from that of skin infections.

Patients with major burns differ from other critically ill patients in that the magnitude of their early exudative trace element losses causes negative balances [7,17] and early deficiencies involving copper, iron, selenium, manganese, and zinc. Such deficiencies have been described recurrently since the 1960s [13,23]. Copper is involved in wound healing (essential for the synthesis of collagen and elastin), immunity (neutrophil function and immunoglobulin synthesis), and antioxidant defences (copper-zinc superoxide dismutase) [24]. Zinc is virtually universal, being involved and essential in nearly every step of anabolism, tissue repair, immunity, endocrine system, and antioxidation [13]. The doses provided by the supplements in our two trials were calculated to provide a little more than substitution. The benefit of the intervention is probably the result of the three trace elements, and not of selenium only. Patients with major burns further differ from other critically ill patients by having lower mortality rates [19], with only three deaths occurring among 41 patients (7.3%). Mortality attributable to VAP is controversial though [25]. The mortality rate of critically ill patients developing VAP may be more directly linked to the underlying condition. This consideration may also apply to burns; indeed, the observed mortality is exactly as predicted by the Ryan score. In addition, combined trace element deficiencies do contribute to altered immune defences in burns; the triple-supplement addressed this particular condition.

Selenium deserves special consideration among the three elements. Animal studies have shown that pre-injury selenium deficiency aggravates the oxidative stress caused by burn injury [26]. In addition, an analogy can be found with other critical illnesses; low selenium status is present in nearly every septic ICU patient [27]. Indeed, this may be specific to Europe, a geographic area characterised by a suboptimal selenium status in the general population [28,29], whereas other trace elements are generally normal. Selenium deficiency is aggravated by acute illness [27]. Therefore, part of our findings may apply to other European critically ill patients, and selenium supplementation may find a place in a multimodal pneumonia prevention strategy, but this must be verified.

## Conclusion

This aggregation study shows that trace element supplementation is associated with a significant reduction of pulmonary infectious complications, mainly due to a reduction of nosocomial pneumonia in critically ill, burned patients. This was associated with a highly significant reduction of the length of ICU stay normalised for burned BSA. The likely underlying mechanism is a reinforcement of endogenous antioxidant defences. The implications of this finding for the management of burned patients are substantial; hence, a larger multicentre trial is required to confirm this preventive effect and to explore its applicability to other critical care conditions.

## Key messages

• Patients with burns on more than 20% of BSA suffer trace element deficiencies, decreased antioxidant capacity, and depressed immunity and are particularly prone to develop infectious complications involving the lung and the wounds.

• Trace element supplementation was associated with a significant reduction of nosocomial pneumonia and of VAP after major burns.

• Reduction of nosocomial pneumonia was associated with a significant reduction of days of antibiotherapy and reduction of length of stay in the ICU normalised for burned BSA.

• A reinforcement of endogenous antioxidant defences is a likely mechanism, considering the observed parallel increases in plasma selenium concentration and GSHPx activity.

• The doses provided by the supplements were calculated to provide a little more than substitution of exudative losses, which is important in Europe, where the general population is characterised by a suboptimal selenium status. Therefore, our findings may apply to other ICU diagnostic categories.

## Abbreviations

BAL = bronchoscopic bronchoalveolar lavage; BSA = body surface area; CHUV = Centre Hospitalier Universitaire Vaudois (Lausanne, Switzerland); GSHPx = glutathione peroxidase; ICU = intensive care unit; PaO_2 _= partial pressure of oxygen; SIRS = systemic inflammatory response syndrome; VAP = ventilator-associated pneumonia.

## Competing interests

The authors received funding from Fresenius Kabi AG (Bad Homburg, Germany) and Laboratoires Aguettant (Lyon, France) to support pharmacy preparation of the intervention solutions and partial laboratory costs. Fresenius Kabi AG reimbursed travel expenses from Geneva to Frankfurt, Germany, for a scientific meeting.

## Authors' contributions

MMB conceived of and designed the study and performed clinical investigation, data collection, data analysis, and manuscript preparation. PE, DKH, and AD performed data analysis and manuscript preparation. RLC conceived of and designed the study and performed data analysis and manuscript preparation. J-PR conceived of and designed the study and performed interpretation of data, data analysis, and manuscript preparation. WR conceived of and designed the study and performed clinical investigation, interpretation of data, and manuscript preparation. AS conceived of and designed the study and performed development of analytical methods, data analysis, and manuscript preparation. All authors read and approved the final manuscript.

## Figures and Tables

**Table 1:** Comparative methods and endpoints of the two supplementation studies

	Study 1 [15]	Study 2 [16]
Methods		
Study design	Prospective, randomised, placebo-controlled trial	Prospective, randomised, placebo-controlled trial
Stratification	None	Age (≥ or <50 years) Inhalation (yes/no) Burned BSA (≥ or <50%)
Inclusion and exclusion criteria	Identical	Identical
Trace elements per day (intravenous)	Copper 40.4 μmol (2.5 mg)/day Selenium 2.9 μmol (315 μg)/day Zinc 407 μmol (26.2 mg)/day	Copper 47.6 μmol (3.1 mg)/day Selenium 4.8 μmol (380 μg)/day Zinc 574 μmol (31.4 mg)/day
Duration of supplementation	8 days	14 days if burns <60% of BSA 21 days if burns ≥60% of BSA
Nutritional management	Early enteral feeding (within 12 hours of admission) targeted at 1.3 times of resting energy expenditure, reached during a period of 4 days	Identical
Vitamins per day	Vitamin C 1 g, vitamin E 100 mg, vitamin B 100 mg, and multivitamin (Cernevit^®^; Baxter, Plessis, France)	Identical
Blood sampling	Days 0, 1, 5, 10, 15, 20, and 30 for plasma trace elements + vitamin dosages	Identical + plasma GSHPx activity
Skin biopsies	None	Days 3, 10, and 20: tissue selenium, zinc, and GSHPx activity
Endpoints		
Clinical endpoints	Length of mechanical ventilation Length of ICU stay Length of ICU stay per burned percentage of BSA	Identical
Wound healing	Success of skin grafting (percentage of grafted area per percentage of area with surgical burns)	Identical + Whole body turnover of glycerol, glucose, and phenylalanine Phenylalanine skin incorporation
Immune response	Chemotaxis capacity of neutrophil T lymphocyte and neutrophil counts Cell surface markers on lymphocytes Adhesion molecules on neutrophils	Not performed
Infectious complications and definition	Prospective surveillance during the first 30 days of stay according to predefined criteria [18]	Identical
Antibiotic treatment	Details on antibiotic delivery (type, dose, and route)	Identical

BSA, body surface area; GSHPx, glutathione peroxidase; ICU, intensive care unit.

**Table 2:** Patient characteristics

	Study 1			Study 2			Study 1 versus Study 2
	(*n *= 20)			(*n *= 21)			
	Supplemented group	Placebo group	Total	Supplemented group	Placebo group	Total	*P *value^a^

	(*n *= 10)	(*n *= 10)	(*n *= 20)	(*n *= 11)	(*n *= 10)	(*n *= 21)	

Age (years)	39.4 ± 15.8	42.6 ± 13.9	41.0 ± 14.6	46.3 ± 15.2	38.4 ± 16.2	42.5 ± 15.8	0.75
Burned BSA percentage	51.5 ± 22.5	44.9 ± 9.7	48.2 ± 17.2.	44.9 ± 22.3	44.3 ± 20.2	44.6 ± 20.8	0.55
Inhalation injury	2 (20%)	5 (50%)	7 (35%)	5 (46%)	4 (40%)	9 (43%)	0.75
SAPS II score	29.0 ± 6.4	27.2 ± 10.0	28.1 ± 8.3	34.3 ± 8.2	32.4 ± 9.3	33.4 ± 8.6	0.052
Ryan score	0.9 ± 0.8	1.0 ± 0.8	1.0 ± 0.8	1.1 ± 1.0	0.9 ± 0.7	1.0 ± 0.9	0.85
Patients on mechanical ventilation for >24 hours	8 (80%)	8 (80%)	16 (80%)	10 (90%)	9 (90%)	19 (90%)	0.34

^a^Studies compared by independent *t *test for continuous data and Fisher exact test for categorical data. BSA, body surface area; SAPS II, Simplified Acute Physiology Score.

**Table 3:** Clinical outcomes

	Supplemented group,	Placebo group,	*P *value^a^
	median (range)	median (range)	

Length of mechanical ventilation (days)	5 (0 to 28)	12 (0 to 28)	0.28
Length of antibiotherapy (days)	13 (3 to 30)	20 (6 to 29)	0.021
Length of ICU stay (days)	28 (9 to 151)	39 (16 to 145)	0.18
Length of ICU stay per proportion of burned BSA (days per percentage of BSA)	0.63 (0.23 to 1.64)	0.99 (0.43 to 2.48)	0.002
Mortality	2 out of 21	1 out of 20	0.57

^a^By stratified log-rank test. BSA, body surface area; ICU, intensive care unit.

**Table 4:** Infectious complications

Type of infection	Supplemented group (*n *= 21) Number of patients per number of episodes (mean episodes ± SD per patient)	Placebo group (*n *= 20) Number of patients per number of episodes (mean episodes ± SD per patient)	P value^a^
Any infection	21/43 (2.0 ± 1.0)	20/69 (3.5 ± 1.2)	<0.001
Pneumonia			
Any	11/13 (0.6 ± 0.7)	20/35 (1.7 ± 1.1)	0.001
Early (0 to 48 hours)	6/6 (0.3 ± 0.5)	4/4 (0.2 ± 0.5)	ns to 0.220
Nosocomial (>48 hours)	7/7 (0.33 ± 0.5)	16/31 (1.55 ± 1.0)	<0.001
VAP^b^	6/6 (0.33 ± 0.5)	13/13 (0.65 ± 0.5)	0.023
Recurrent^c^	2/2 (0.1 ± 0.3)	13/19 (0.95 ± 0.8)	<0.001
Skin and soft tissue infection	14/20 (0.95 ± 0.9)	14/19 (0.95 ± 0.8)	ns to 0.871
Urinary tract infection	3/4 (0.2 ± 0.5)	4/5 (0.253 ± 0.6)	ns to 0.726
Bloodstream infection	6/6 (0.3 ± 0.5)	5/7 (0.35 ± 0.7)	ns to 0.734
Other infection^d^	0	4/4 (0.2 ± 0.4)	ns to 0.031

^a^*P *values are generated from Cochran-Armitage trend test; ^b^VAP reduced from 5.5 to 3.6 episodes per 1,000 ventilator days in supplemented patients; ^c^recurrent pneumonia designates new distinct pneumonia occurring after a first episode of pneumonia (early or nosocomial); ^d^including three cases of enterocolitis and one case of chondritis of the ear. NP, nosocomial pneumonia; ns, non-significant; SD, standard deviation; VAP, ventilator-associated pneumonia.
